# Nutritional ketosis as an intervention to relieve astrogliosis: Possible therapeutic applications in the treatment of neurodegenerative and neuroprogressive disorders

**DOI:** 10.1192/j.eurpsy.2019.13

**Published:** 2020-01-31

**Authors:** Gerwyn Morris, Michael Maes, Michael Berk, André F. Carvalho, Basant K. Puri

**Affiliations:** 1 Deakin University, IMPACT Strategic Research Centre, Barwon Health, School of Medicine, Geelong, Victoria, Australia; 2 Department of Psychiatry, Chulalongkorn University, Faculty of Medicine, Bangkok, Thailand; 3 Deakin University, CMMR Strategic Research Centre, School of Medicine, Geelong, Victoria, Australia; 4 Orygen, The National Centre of Excellence in Youth Mental Health, The Department of Psychiatry and the Florey Institute for Neuroscience and Mental Health, University of Melbourne, Parkville, Victoria, Australia; 5 Department of Psychiatry, University of Toronto, Toronto, Ontario, Canada; 6 Centre for Addiction and Mental Health (CAMH), Toronto, Ontario, Canada; 7 C.A.R., Cambridge, England, United Kingdom

**Keywords:** Astrogliosis, ketosis, neurodegeneration, neuroprogression, molecular neurobiology

## Abstract

Nutritional ketosis, induced via either the classical ketogenic diet or the use of emulsified medium-chain triglycerides, is an established treatment for pharmaceutical resistant epilepsy in children and more recently in adults. In addition, the use of oral ketogenic compounds, fractionated coconut oil, very low carbohydrate intake, or ketone monoester supplementation has been reported to be potentially helpful in mild cognitive impairment, Parkinson’s disease, schizophrenia, bipolar disorder, and autistic spectrum disorder. In these and other neurodegenerative and neuroprogressive disorders, there are detrimental effects of oxidative stress, mitochondrial dysfunction, and neuroinflammation on neuronal function. However, they also adversely impact on neurone–glia interactions, disrupting the role of microglia and astrocytes in central nervous system (CNS) homeostasis. Astrocytes are the main site of CNS fatty acid oxidation; the resulting ketone bodies constitute an important source of oxidative fuel for neurones in an environment of glucose restriction. Importantly, the lactate shuttle between astrocytes and neurones is dependent on glycogenolysis and glycolysis, resulting from the fact that the astrocytic filopodia responsible for lactate release are too narrow to accommodate mitochondria. The entry into the CNS of ketone bodies and fatty acids, as a result of nutritional ketosis, has effects on the astrocytic glutamate–glutamine cycle, glutamate synthase activity, and on the function of vesicular glutamate transporters, EAAT, Na^+^, K^+^-ATPase, K_ir_4.1, aquaporin-4, Cx34 and K_ATP_ channels, as well as on astrogliosis. These mechanisms are detailed and it is suggested that they would tend to mitigate the changes seen in many neurodegenerative and neuroprogressive disorders. Hence, it is hypothesized that nutritional ketosis may have therapeutic applications in such disorders.

## Introduction

**Figure 1.**
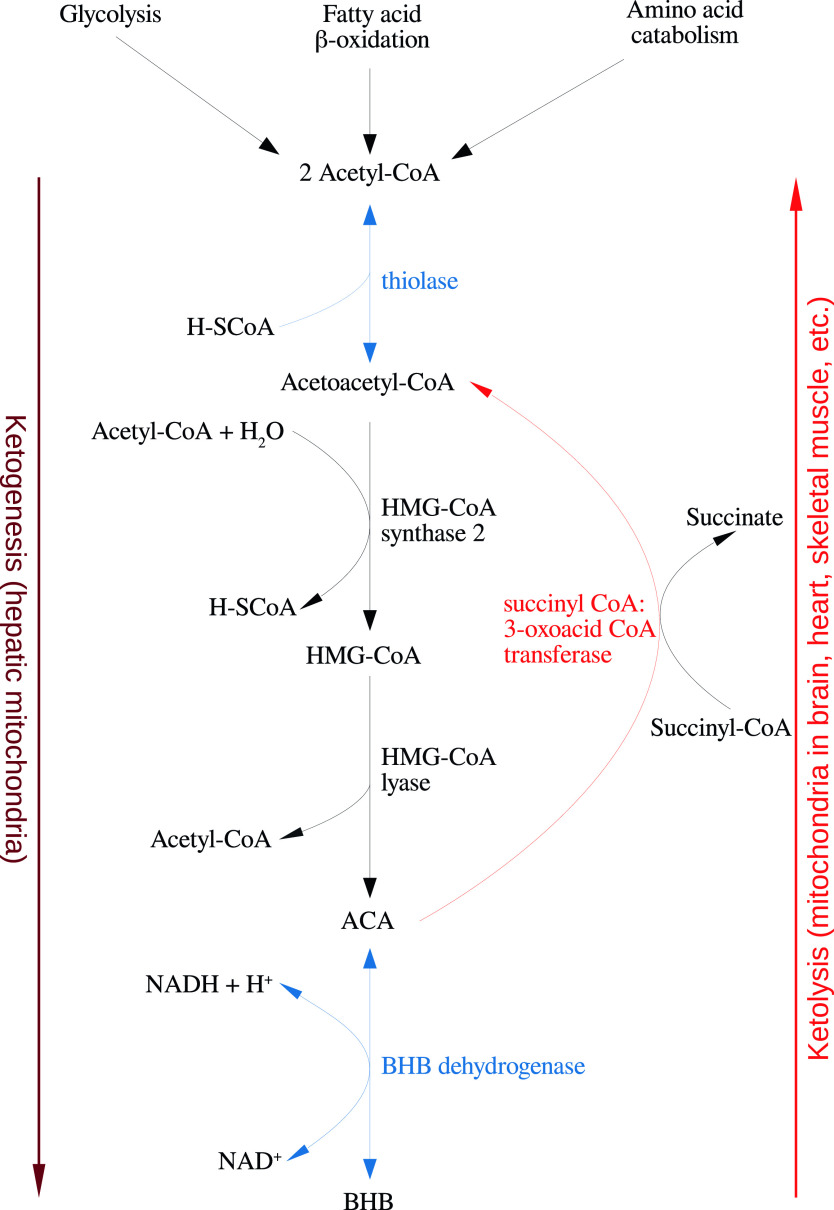
Summary of the reactions of ketogenesis and ketolysis. Abbreviations: ACA, acetoacetate; BHB, β-hydroxybutyrate.

Several nutritional approaches are now available to clinicians wishing to induce ketosis in their patients in the periphery and/or the brain in order to further positive therapeutic outcomes and the details of such approaches are usefully reviewed in [1] and [2] and depicted in Figure 1. A state of induced ketosis via the classical ketogenic diet (KD), the modified KD, or the medium-chain triglyceride (MCT) diet have long been successful therapeutic interventions in the treatment of many children with pharmacologically resistant epilepsy and the efficacy of these diets have been confirmed in large studies [3,4]. More recently, the results of prospective studies and meta-analyses have also confirmed the efficacy of these diets in the treatment of intractable epilepsy in adults [5,6]. There is also some evidence to suggest that the modified Atkins diet may have efficacy irrespective of patient age [7,5].

Unsurprisingly, there has been considerable interest in the putative therapeutic utility of dietary ketosis as a possible treatment approach for neurological and neuropsychiatric (increasingly described as neuroprogressive) illnesses which are very often refractory to current standard pharmaceutical interventions. In this context, it is noteworthy that some research teams investigating this area have reported some success most notably in patients with mild cognitive impairment or early Alzheimer’s disease (AD) [8]. This is also true of interventions based on elevating levels of β-hydroxybutyrate (BHB), which is one of the molecules thought to underpin many of the therapeutic benefits of the classical and modified KD [8]. However, it must be emphasized that this latter approach does not induce ketosis but rather a state described as ketonemia. The different biochemistry involved in these two states is explained in an excellent review by Reger et al. [9].

Importantly, positive results have been reported using a wide range of methods now available for inducing a state of ketosis in animals and humans such as an emulsified MCT diet [10], oral ketogenic compound [11], fractionated coconut oil [12], very low carbohydrate diet [13], and a ketone monoester dietary supplement [14]. There are also encouraging data suggesting that a KD might benefit individuals with Parkinson’s disease (PD) [15]. There is also some evidence to suggest that nutritional ketosis might benefit patients with schizophrenia (SZ) [16], bipolar disorder (BPD) [17], and autistic spectrum disorder (ASD) [17]. However, a literature search by the authors did not to reveal any studies which examined the effect of the KD on patients with major depressive disorder (MDD), which is curious given the existence of data demonstrating a positive effect of the diet, or its variants, on tryptophan metabolism [18], as abnormal tryptophan metabolism is considered to be involved in the pathogenesis of the illness [19,20]. In addition, MDD patients have higher levels of pro-inflammatory cytokines (PICs) [21] and hence a KD can be viewed as a potential treatment for MDD because it has an anti-inflammatory property [18]. In this context, it is noteworthy that commonly used antidepressants have anti-inflammatory activity [22] while anti-cytokine agents can improve anhedonia [23]. Furthermore, KD increases the levels of brain-derived neurotrophic factor (BDNF) [24]. Similarly, novel antidepressants also increase BDNF levels in the hippocampus [25,26]. As a result, the KD has the potential to modulate neurotrophic pathways and inflammatory mechanisms to reduce depressive symptom severity and other dimensions of depressive psychopathology including cognition [18]. The use of a KD in MDD, and indeed other neuroprogressive conditions, is also supported by data gleaned from animal studies, and readers interested in the area are invited to consult an excellent review by Fabrazzo [27]. Unsurprisingly, there has been a plethora of research investigating the mode of action of nutritional ketosis and how it produces its therapeutic effects, and certain themes have emerged.

For example, nutritional ketosis and the influx of ketone bodies (KBs) and medium-chain fatty acids (MCFAs) into the brain provide an alternative source of energy to glucose and exert a glucose sparing effect in the brain in an environment of glucose restriction or relative hypometabolism thereby allowing the preservation or even improvement of brain function and neuronal survival [2, 28–31]. This appears to be potentially of considerable therapeutic potential as far as the treatment of neurodegenerative and neuropsychiatric disorders, henceforth described as neuroprogressive, disorders is concerned, as glucose hypometabolism is observed in AD [32], PD [33], amyloid lateral sclerosis (ALS) [34], Huntington’s disease (HD; [35]), SZ [36], BPD [37] and MDD [38]. The therapeutic importance of addressing the relative glucose hypometabolism and its consequences in patients suffering from neurodegenerative or neuroprogressive disorders are emphasized by data suggesting that this state plays a causative role in the pathogenesis of these illnesses and in some cases may be observed long before symptoms or other recognized drivers of pathology are apparent [39].

In addition, there is accumulating preclinical and clinical evidence that dietary ketosis results in the amelioration of oxidative stress, mitochondrial dysfunction and inflammation in the periphery, and in the brain of animals and humans [40–46]. Such data may also have therapeutic relevance as the weight of evidence strongly suggests that oxidative stress and mitochondrial dysfunction have a causative role in the pathogenesis of neurodegenerative [47,48] and neuroprogressive [49,50] disorders.

Although much of the research in neurodegenerative and neuroprogressive diseases has focused on the detrimental effects of oxidative stress, mitochondrial dysfunction and neuroinflammation on neurone function [51,52], and survival, there is now a growing appreciation that this triad of abnormalities exerts pathology by compromising neurone–glial cell interactions and disrupting the normal roles played by microglia and astrocytes in central nervous system (CNS) homeostasis [53,54]. Mitochondrial dysfunction in astrocytes and microglia is of particular pathological significance from the perspective of compromised homeostasis in the CNS as the regulatory functions of microglia and astrocytes are dependent on optimal mitochondrial function and the maintenance of these glial cells in their physiological state [55–57].

In addition, while disturbed mitochondrial function impairs the ability of microglia and astrocytes to regulate multiple dimensions of CNS homeostasis, the same is true of raised levels of oxidative stress via mechanisms independent of induced mitochondrial dysfunction. In brief, elevated levels of reactive oxygen species (ROS) and reactive nitrogen species (RNS), and/or compromised cellular antioxidant systems sensitize microglia to activation by inflammatory mediators and hence exacerbate levels of inflammation and promote a variable state of reactivity and dysfunction described as astrogliosis [58,59].

The development of astrogliosis leads to impairment or loss of homeostatic functions of these glial cells in regulating brain homeostasis [60]. This is highly problematic from the perspective of brain function and is considered to be a critical event in the pathogenesis and pathophysiology of neurodegenerative and neuroprogressive illnesses as accumulating data strongly suggest that such a reactive and dysfunctional state in astrocytes is a major if not the main driver of neural dysfunction or neurodegeneration seen in these illnesses [61,62].

Unsurprisingly, given the evidence discussed above, the modulation of astroglial activity and function is now considered to be an important therapeutic target in the treatment of neurodegenerative and neuroprogressive diseases [60, 63–65]. From this perspective, it is encouraging that several authors have reported that induced ketosis decreases astrocyte activity, improves astrocyte–neurone interactions [66,67], and exerts positive effects on expression and function of receptors-enabling astrocytes to regulate multiple dimensions of CNS homeostasis [29, 68–71].

Clearly, there is accumulating evidence suggesting that the use of nutritional ketosis may result in a beneficial manipulation of astrocyte activity and function. However, there appear to be few publications relating to dietary ketosis exclusively focusing on this topic. Hence, this article aims to address this apparent gap in the literature by attempting to explain the various biochemical and energetic consequences of dietary ketosis from the perspective of microglia and astrocytes. In order to facilitate this endeavor, we will first outline the processes involved in the generation of a ketotic state before discussing entry of KBs and fatty acids (FAs) into the brain and the consequences of such entry on energy production, cellular antioxidant defences, and levels of neuroinflammation. We will then move on to consider the elements driving astrogliosis and its functional consequences before focusing on the potential remedial effects of nutritional ketosis on the disturbed patterns of astroglial function seen in neurodegenerative and neuroprogressive conditions.

## The Biochemistry of Ketogenesis

Under physiological conditions, acetyl CoA produced by FA oxidation enters the tricarboxylic acid (TCA) cycle and subsequently engages in a chemical reaction with oxaloacetate to produce citrate. However, under metabolic conditions induced by the KD, oxaloacetate is exported out of the mitochondria, being utilized for the process of gluconeogenesis [72]. In this scenario, levels of acetyl CoA synthesis greatly exceed the amount of oxaloacetate in the mitochondrial environment and the former engages in a series of condensation reactions, which are the hallmark of ketogenesis [73]. First, two acetyl CoA molecules combine to produce acetoacetyl CoA. This molecule reacts with a further molecule of acetyl CoA to form HMG-CoA in a functionally irreversible and rate limiting reaction enabled by HMG-CoA synthase 2 [74]. Once formed, this compound dissociates to the KB acetoacetate (ACA), which is further reduced to BHB by a reaction enabled by BHB dehydrogenase and involving the NAD^+^/NADH couple as the hydrogen donor [75]. It should be noted that levels of BHB in the circulation and tissues are much higher than those of ACA, making the former the predominant KB [76,77].

BHB and ACA are exported into the circulation from the liver and ultimately imported by the brain, heart, skeletal muscle, and other tissues with high metabolic demands [73]. Once ensconced in these body compartments, BHB is oxidized to ACA by BHB dehydrogenase, which acts as a prime regulator of the mitochondrial NAD^+^/NADH ratio status [78]. ACA is then hydrolyzed to form acetoacetyl CoA and succinate in a reaction enabled by the enzyme succinyl CoA:3-oxoacid CoA transferase, and the acetoacetyl CoA is then cleaved to yield acetyl CoA in a reaction catalyzed by thiolase; the acetyl CoA and succinate form substrates for the TCA cycle and complex II of the electron transfer chain (ETC), respectively [79]. This process may also result in increased succinate dehydrogenase activity reported following prolonged administration of the KD in rodents [80]. These pathways are depicted in Figure 1. The effects of the KD may be mimicked by the use of KB supplements and there is at least some evidence to suggest that the production of KBs in the liver, which occurs in physiological conditions may be inhibited in such a scenario although this is not universally accepted [9,81].

KBs are metabolized at a considerably higher rate than glucose and enter the TCA cycle directly as previously discussed, thus bypassing glycolysis [77,82]. Importantly, much evidence suggests that at levels normally induced by ketogenesis, glycolytic ATP generation diminishes and the generation of ATP by oxidative phosphorylation increases [83,84]. Although the β-oxidation of free fatty acids (FFAs) is clearly a factor underpinning such observations, other mechanisms are also involved and we turn to a consideration of these elements in the next section of this article.

## Entry of Ketone Bodies and FAs into the Brain

When plasma KB concentration exceeds 4 nM, the uptake of these molecules into the brain increases ([85,86]; reviewed [87]). Several research teams using in vivo PET techniques have reported a magnitude of increase in brain KB concentrations induced by a prolonged KD in humans and rodents of approximately eightfold compared with controls fed a normal diet [31,85,86]. However, the extent of ketosis is of importance as experimental evidence suggests that mild ketosis only produces a doubling of KB levels in the brain [88].

Plasma KB levels are also of importance because such levels are proportional to increased KB levels and metabolism in the brain, which in turn determine the global degree of KB-induced glucose metabolism suppression within the CNS [31]. Evidence suggests that the suppression of glucose metabolism in the CNS induced by KBs increases by approximately 9% for every 1 nM increase in KB levels in the plasma [30,89]. The importance of KBs as an energy source in conditions of ketosis induced by diet or starvation is graphically illustrated by the presence of data demonstrating that these molecules may supply approximately 60–70% of the brain’s energy needs in such conditions [90].

KBs and MCFAs (produced from MCT supplementation in some versions of the KD, as discussed above) transverse the blood–brain barrier (BBB) into the brain via the assistance of monocarboxylate 1 and 2 transporters expressed on brain microvascular endothelial cells [91,92]. The expression of these transporters increases over 10-fold following a protracted period of ketosis [93]. Polyunsaturated fatty acids (PUFAs) can also cross the BBB although the enabling mechanisms are a matter of debate and the assistance of calveolin-1, FA transporters, phospholipid-bound FA translocase, and lipoprotein packaging have all been posited (reviewed by [75]). However, current evidence suggests that long-chain nonesterified FAs (NEFAs) cannot cross the BBB at a sufficient rate to meet energy demands [94].

## Consequences of Ketogenesis and Ketolysis in the CNS

Dietary induced ketosis is associated with increased ATP levels in the brain [95–99]. Other in vivo effects include increased phosphocreatine [100,101] and increased ATP synthase [100,102]. These changes are associated with increased numbers of mitochondria [95,100] and improved levels of mitochondrial performance in glia [103] and neurones [104]. It is important to note that this pattern of globally increased metabolism is observed in patients with AD following ingestion of the MCT diet and thus there is good reason to believe that these effects would also occur in patients suffering from other neurological and indeed neuroprogressive disorders [105].

There is also a significant and accumulating body of evidence demonstrating a statistically significant reduction in oxidative and nitrosative stress and upregulation of cellular antioxidant defences in the brains of animals following prolonged dietary ketosis ([106–111]; reviewed by [41]). This decrease would also appear to be clinically significant as several authors have reported a reduction in oxidative damage to neurones and increased neuronal survival as a result of dietary induced ketosis especially in an environment of cerebral glucose deprivation [109, 111–113].

Several mechanisms appear to underpin the reductions in CNS oxidative stress induced by the KD, with ROS scavenging by KBs being the simplest. Another route involves the maintenance of ETC performance, particularly complexes I, II, and III resulting in reduced ROS production by mitochondria [40,109,111,112].

Ingestion of KDs also leads to upregulation of Nrf2 in the brain [43,45,46]. This is of paramount importance as activation of this transcription factor activates a myriad of cellular antioxidant enzymes and nonenzymatic elements of the cellular antioxidant response system. The cellular antioxidant enzymes include superoxide dismutase, catalase, thioredoxin reductase, glutathione peroxidase, glutathione transferase, glutathione reductase, and the peroxidase family [114,115]. The nonenzymatic elements include carbon monoxide, thioredoxin, and reduced glutathione (GSH) [116].

Nutritional ketosis and increased levels of KB can also activate a plethora of other transcription factors and increase levels of several molecules, which can activate many signaling pathways resulting in reduced oxidative stress and metabolic adaptation to energy production via FA oxidation and ketolysis, which also have the effect of reducing mitochondrial ROS generation (reviewed by [117]). For example, the weight of in vivo data associates dietary-induced ketosis with elevated levels and activity of AMP-activated protein kinase (AMPK) in the brain in rodents and humans [118,119]. In vitro data suggest that such upregulation in astrocytes occurs to a much greater degree in these glial cells than neurones [120].

Prolonged ketosis is also associated with upregulation of NAD^+^ [75,82,121,122]. Increased levels of NAD^+^ explain the upregulation of the histone deacetylases sirtuin-1 (SIRT-1) and SIRT-3 seen in the brains and peripheral tissues of animals fed a KD, as SIRTs are NAD^+^-dependent enzymes [123–125].

There exist reports of FOXO3a, PGC-1α, and PPARγ elevation in animals fed a KD [42,126,127]. This is consistent with the work of several other authors who have reported that the upregulation of these transcription factors is driven by the upregulation of NAD^+^, AMPK, and SIRTs via a number of different routes [1,128]. The upregulation of these molecules also leads to activation of Nrf2 [117], which provides another route for activation of this transcription factor in addition to increases in NO and ROS levels [129].

The activation of the cascade described above results in increased cellular antioxidant systems and a downregulation of oxidative stress together with improved mitochondrial performance generation and a series of long-term metabolic adaptations designed to improve the efficiency of FA oxidation via mechanisms described in [130]. Clearly, the activation of signaling pathways subsequent to KD activation of AMPK, NAD^+^, and SIRTs explains the beneficial effects of dietary induced ketosis on ATP production, mitochondrial function, and oxidative stress in the brain, which are all therapeutic targets as far as the treatment of neuroprogressive and neurodegenerative diseases is concerned.

However, the data supplied by several research teams describing the activation of PPARs in the CNS of animals following KD ingestion appear worthy of particular focus from the perspective of this article for a number of reasons [127,131,132]. The first stems from the fact that PPARα and PPARγ are the main transcription factor-regulating ketogenesis and ketolysis and both PPAR isoforms are activated by increased levels of FFAs in the periphery and brain, a few days after the advent of ketosis [75,133,134]. The second is that several authors have reported that the upregulation of PPAR isoforms in the brain results in a reduction in neuroinflammation in vivo [125,126,135,136]. The third is that PPAR upregulation has the capacity to rescue mitochondrial dysfunction in the CNS environment typical of neurodegenerative [137] and neuroprogressive disorders [137,138].

Importantly, these effects extend to astrocytes. For example, the in vivo reduction in increased PPAR levels in astrocytes reduces mitochondrial dysfunction, decreases inflammation, and increases cellular antioxidant defences [139–141]. These data are also of importance from the perspective of improving astrocyte function as all these factors are also involved in driving and maintaining a reactive state in these glial cells [142].

Unsurprisingly, given the data discussed above, there are also an accumulating number of in vivo and in vitro studies where the authors report beneficial changes to astrocyte functions including improved glutamate and potassium homeostasis via direct effects on surface receptors [143,144]. There is also a growing awareness that many of these effects ultimately arise from the effects of ketone bodies and FAs translocated from the periphery on astrocyte metabolism and the involvement of these glial cells in mediating de novo ketogenesis in the brain. We will now move on to discuss these elements beginning with the role of astrocytes in FA oxidation and energy production.

## Role of Astrocytes in Energy Production and Distribution

Astrocytes are regarded as the main site of FA oxidation in the brain [145,146]. There is also an accumulating body of evidence to support the view that astrocyte-derived KBs produced by FA oxidation in an environment of glucose restriction can be a significant source of oxidative fuel for neurones [147–150]. Indeed, there is a developing consensus that KBs supplied by astrocyte-mediated FA oxidation rather than KBs translocated from the periphery are the dominant source of these molecules in the brain in a state of ketosis [151,152]. This phenomenon is of paramount importance as far as neurone function and survival in an environment of glucose hypometabolism is concerned as the so-called lactate shuttle between astrocytes and neurones, which provides the oxidative substrate for neurones in physiological conditions, becomes compromised in such an environment owing to its dependence on glycogenolysis and glycolysis [153,154].

The reason for the dependence of the lactate shuttle on glycolysis stems from the fact that the astrocyte filopodia responsible for the release of lactate, either via monocarboxylate transporters or by passive diffusion, is too narrow to accommodate mitochondria [153,154]. There are numerous papers discussing the evidence confirming the existence of an astrocyte–neurone lactate shuttle in physiological conditions and detailing the mechanisms underpinning its operation and hence it will not be considered further here. Readers interested in an in-depth treatment of this phenomenon are referred to an excellent review by Zhang et al. [155].

In vitro experiments have produced conflicting results regarding the usage or otherwise of MCFAs as a substrate for FA oxidation most notably with regard to octanoate where authors have either reported that astrocytes do not appear to utilize this FA as a substrate for KB production [156]. Another research team reported a twofold increase in KB production following octanoate addition and a 50% increase in the production of lactate following the addition of decanoate to the culture medium [157]. However, the weight of in vivo evidence is consistent with the latter findings as several authors have reported significantly increased KB production by astrocytes following assimilation of MCFAs translocated across the BBB [158–160].

However, it is worthy of note that the preferred FA substrates of astrocytes in the hippocampus may be different [160] and there is some evidence to suggest that even long-chain FAs may be utilized in some circumstances despite their relative mitotoxicity [160]. Interestingly, data suggest that the relative inhibitory effects of MCFAs on oxidative phosphorylation [161] may increase KB production in astrocytes and improve the efficiency of the astrocyte–neurone shuttle [157], although these observations could also be explained by the inhibitory effect of MCFAs on glycolysis and the resultant improvement in the efficiency of ketogenesis [161]. Finally, it should be noted that MCFAs are not the only substrates enabling KB production in astrocytes as these glial cells may utilize branched-chain amino acids such as leucine for this purpose in certain circumstances [162]. Similarly, not all KB production by astrocytes is destined for neurones as an appreciable amount is used for cholesterol and lipoprotein synthesis [145].

Having discussed the role of astrocytes in ketogenesis and ketolysis in the brain, we now move on to consider how ketogenesis positively modulates the metabolism, signal transduction, and receptor profiles of astrocytes, which may mitigate against the neuropathological consequences of reactive astrogliosis. However, before doing so, it is necessary to explain the origins and consequences of this phenomenon.

## Causes and Consequences of Astrogliosis

### Causes of astrogliosis

Astrocytes are exquisitely sensitive to very small fluctuations in the CNS intracellular environment and readily attain a reactive phenotype in the face of such changes. One acknowledged cause of increased astrocyte reactivity is increased intracellular levels of PICs, NO, and ROS secreted by activated microglia [163–165]. Other triggers include glucose deprivation, increased levels of ATP and other gliotransmitters, and activation of surface toll-like receptors by commensal lipopolysaccharide (LPS) originally translocated from the intestine. It is important to note that the development of reactive astrogliosis provoked by these stimuli, particularly the inflammatory mediators such as the PICs, ROS, and NO, is accomplished via major changes in astrocyte gene transcription patterns, which drive morphological, physiological, and biochemical changes ([142]; reviewed by [166]).

Although there are a myriad of changes in signaling pathways in activated astrocytes compared with those existing in these glial cells in their physiological state, from the perspective of this article, the most noteworthy change is the chronic activation of the NF-κB, MAP kinase, and Jak/Stat pathways, which result in high intracellular levels of ROS, RNS, and PICs (reviewed by [167]). It is noteworthy that STAT-3 is of paramount importance as the weight of evidence suggests that activity of this transcription factor is an indispensable element in the development and maintenance of reactive astrogliosis [168–170].

Readers interested in the details of mechanisms underpinning the advent and persistence of a reactive or dysfunctional state in astrocytes are invited to consult the work of [171] and [60]. However, the key points to bear in mind during a perusal of the following sections of this article are that many of the factors underpinning the loss of homeostatic functions normally exerted by astrocytes in their physiological state stem from the changes in transcription orchestrated by the chronic activation of the pathways and transcription factors discussed above and/or the ensuing increases in levels of PICs, ROS, and RNS. These adversely affect the transcription and/or function of crucial membrane receptors and impair mitochondrial respiration and dynamics [59,172,173]. The importance of the latter is difficult to overemphasize as the homeostatic roles of astrocytes depend on adequate performance of mitochondria [55,56,174], and on a wider note, a host of neural–glial interactions also depend on optimal mitochondrial function [175]. Hence, a therapeutic approach, such as the KD, capable of reducing oxidative stress and inflammation in the brain in vivo while improving mitochondrial function has the potential to mitigate against the severity of astrogliosis and improve CNS homeostasis.

### Consequences of astrogliosis

**Figure 2.**
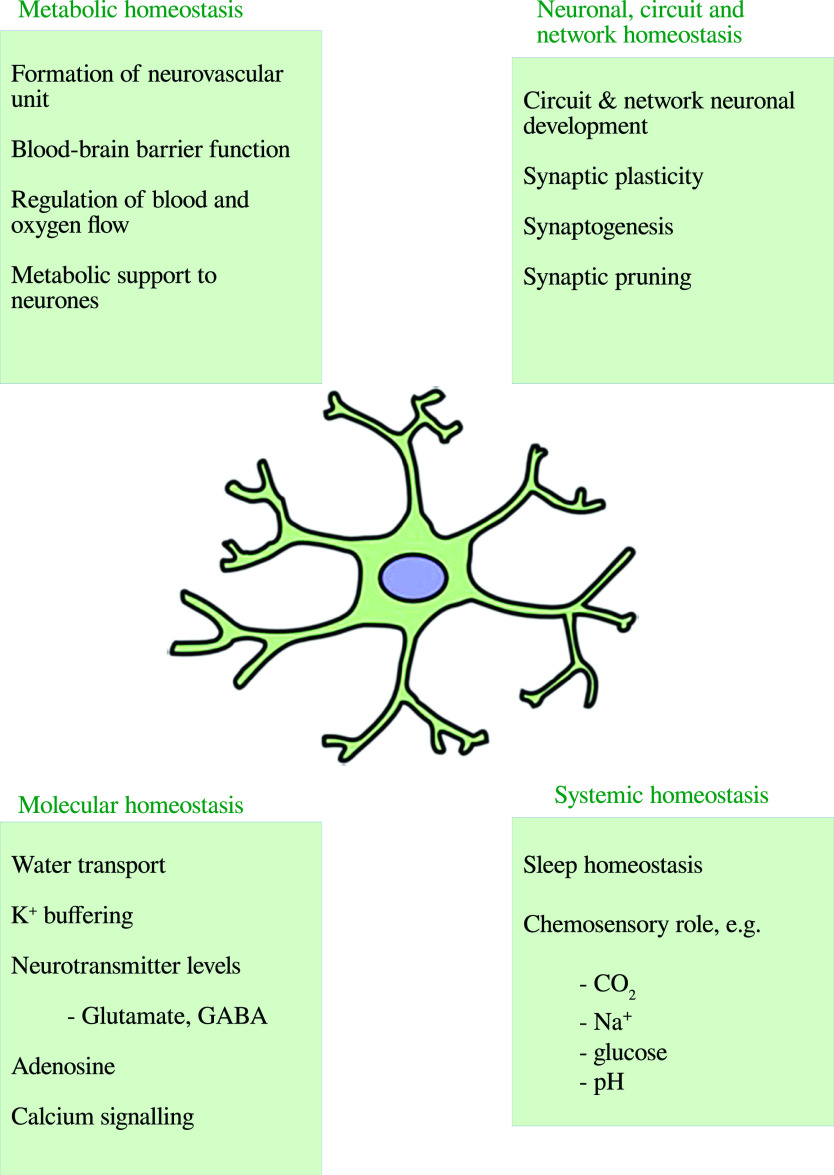
The multiple roles of astrocytes in central nervous system homeostasis.

**Figure 3.**
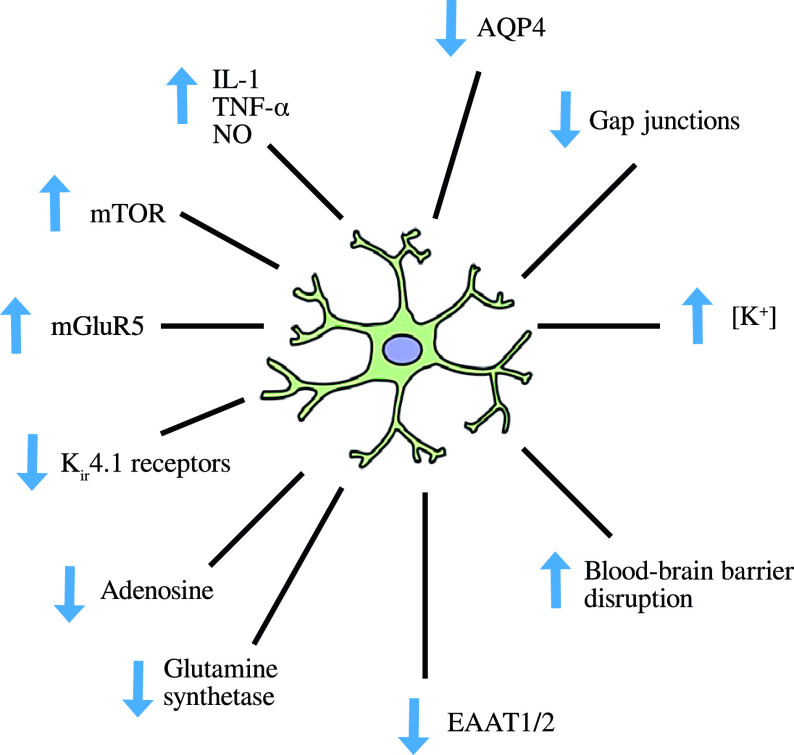
Disruption of central nervous system homeostasis resulting from astrogliosis.

The normal role of astrocytes in regulating other dimensions of CNS homeostasis such as neurotransmitter levels, water transport, waste product clearance, ion homeostasis, and glucose and oxygen delivery to neurones (reviewed by [176] and summarized in Figure 2) are impaired when astrocytes are in a reactive state [177,178]. These observations are underpinned by the fact that astrogliosis results in adverse changes in astrocyte phenotype, signaling pathways, and surface receptor expression, which normally enable these cells to perform their essential role in the regulation of various dimensions involved in the maintenance of CNS homeostasis as described above. Disruption of the neurovascular unit owing to a loss of astrocyte end-feet and other cellular protrusions is perhaps the most damaging physical change as far as CNS homeostasis is concerned. The physical connection between BBB epithelial cells and neurones is needed to deliver nutrients and oxygen to the latter (reviewed by [179]). Examples of changes in receptor levels and function [180] include oxidative modification of glutamate receptors leading to inhibition of astrocyte-mediated glutamate uptake, nitrosylation of the gap junction channel connexin 43 (Cx43), and several gap junction pannexins (reviewed by [181]) leading to dysregulated calcium signaling and ATP transfer between astrocytes and neurones and other astrocytes [182]. Astrogliosis is also associated with profound disturbances in potassium homeostasis as a result of oxidative modification and downregulation of Na^+^, K^+^-ATPase (NKA) [180], and the weak inwardly rectifying K_ir_ family potassium channel K_ir_4.1 [183,184]. The effects of astrogliosis in disrupting CNS homeostasis are summarized in Figure 3.

Given this information, it probably comes as no surprise to learn that astrocyte dysfunction, astrogliosis, and the accompanying global loss of the physiological functions of astrocytes in the regulation of CNS homeostasis play a major role in the development and acceleration of most if not all neurodegenerative [185,186] and neuroprogressive disorders [187,188]. In addition, treating the causes and/or consequences of astrogliosis is a major therapeutic objective [61,63,166]. How that might be achieved, at least in part, via dietary induced ketosis will be discussed in the next section of this article. In order to facilitate this endeavor, we will divide the following section into effects on the glutamate–glutamine cycle, glutamine synthetase, glutamate secretion, glutamate uptake via EAATs, NKA, K_ir_4.1 receptors, aquaporin-4 (AQP4), gap junctions, and K_ATP_ channels.

## Nutritional Ketosis, Astrocyte Functions, and Astrogliosis

### Effect of nutritional ketosis on the astrocyte glutamate–glutamine cycle

There is evidence that the entry of KBs into astrocytes stimulates mitochondrial metabolism and accelerates flux through the TCA cycle resulting in a number of consequences including the upregulation of the glutamate–glutamine cycle ([71,189–191]; reviewed by [144]). The mechanisms underpinning these observations are a matter of debate but stimulation of glycolysis, enhanced efficiency of pyruvate utilization, and increased levels of succinate to overcome a shortfall of oxaloacetate in an environment of glucose restriction all appear to be involved ([74,157,192]; reviewed by [193]).

The weight of evidence suggests that this phenomenon results in increased synthesis of glutamine and upregulated levels of GABA coupled with decreased synthesis of glutamate [71, 189–191, 194, 195], although the latter appears to be highly dependent on astrocyte KB concentration [196]. The existence of the glutamate–glutamine cycle is limited to astrocytes as these glial cells uniquely possess the two enzymes needed for its operation, namely pyruvate carboxylase, which enables the synthesis of new TCA cycle intermediates via the replenishment of oxaloacetate, and glutamine synthetase, which enables the synthesis of glutamine [197,198].

These neurotransmitters must be continually re-synthesized as uptake of GABA by astrocytes is limited and glutamate is used as a substrate for oxidation to compensate for the huge energetic costs of glutamate uptake (reviewed by [199]). Readers interested in the biochemistry underpinning the glutamate–glutamine cycle and the re-syntheses of glutamate and glutamine are invited to consult a comprehensive review on these matters by O'Gorman Tuura et al. [198].

However, there are some key points germane to the discussion below. First, the cycle is highly dependent on energy supplied by glucose oxidation and optimal mitochondrial function [200]. Second, KBs are the preferred oxidative substrate for “powering” the glutamate–glutamine cycle in the environment of cerebral glucose hypometabolism seen in neurodegenerative and neuroprogressive disorders [201]. Third, and perhaps expectedly, evidence of dysfunction or dysregulation of this cycle is present in at least some regions of the brain in patients with SZ [202], MDD [203], BPD [204], AD [205], HD [206], ALS [207], and PD [208]. Moreover, impairment of this cycle is a major element in the development of disturbed glutamate homeostasis, with glutamate excitotoxicity, and reduced GABAergic signaling, which play a causative role in the pathogenesis of most if not all neurodegenerative and neuroprogressive conditions [209,210]. Finally, the weight of evidence suggests that the ultimate source of glutamate and GABA dyshomeostasis seen in these illnesses is the presence of chronic astrogliosis [211,212].

### Nutritional ketosis, astrogliosis, and glutamate synthetase

Astrogliosis is associated with reduced activity of glutamine synthase and GABA synthesis and relative failure of astrocyte-mediated glutamate reuptake as well as disruption of the glutamate–glutamine cycle [211–213]. Astrogliosis is also associated with increased expression of the cysteine/glutamate antiporter channel (X_c_^−^) resulting in increased glutamate signaling and oxidative downregulation of the astrocyte glutamate uptake receptors EAAT1 and EAAT2, which in turn leads to the development of glutamate-mediated *N*-methyl-d-aspartate (NMDA) receptor excitotoxicity [166,214,215]. Impaired glutamate synthetase (GS) activity results in the accumulation of glutamate and impaired glutamate uptake thus making a contribution to the development of excitotoxicity [216].

Several research teams have reported that GS activity in humans and animals is downregulated in a cerebral environment of chronic oxidative and nitrosative stress and that such downregulation results in elevated intracellular glutamate and reduced glutamine levels [217–220]. Moreover, there are replicated in vivo data demonstrating that a major cause of GS inhibition in vivo is nitrosylation or nitration of functional residues secondary to excessive NMDA receptor activity and perhaps even more importantly that in vivo inhibition of extrasynaptic NMDA results in the upregulation of GS activity and increased levels of glutamine [221,222].

This is of interest as a mechanism known to downregulate NMDA activity in vivo is NO-mediated S-nitrosylation of functional thiol groups in NMDA receptor subunits and animal studies have reported raised NO levels in the CNS following consumption of a diet aimed at inducing ketosis [223–225].

It is also noteworthy that ex vivo studies have demonstrated a direct inhibitory effect of MCFAs on NMDA receptor excitotoxicity via the inhibition of NMDA AMPA subunits [226–228] which is of interest as MCT supplemented versions of the KD produced significant benefits in the treatment of patients with AD. Ex vivo data also suggest a positive effect of MCFAs on astrocyte mitochondrial biogenesis (reviewed by [229]).

### Nutritional ketosis, astrogliosis, glutamate release, and synthesis

There are some ex vivo and in vivo data suggesting that KBs suppress the release of glutamate from astrocytes and neurones by inhibiting the actions of vesicular glutamate transporters (VGLUTs) and via an as yet undelineated mechanism [230]. The reduction of glutamate synthesis by BHB in vitro in a dose-dependent manner has also been reported [196]. An in vivo study involving rodents conducted by Olson et al. also appears to be worthy of consideration in the context of the potential beneficial effects of the KD on glutamate and GABA homeostasis. These authors reported that the reduction in glutamate and increase in GABA levels seen in the hippocampus of their study animals was effected by KD-induced changes to the microbiota [231].

### Astrogliosis and EAAT function

However, despite the presence of data suggesting that diet-induced ketosis may have beneficial effects on some drivers of glutamate and GABA dyshomeostasis, it must be noted that there appears to be no direct evidence that a KD exerts positive effects on the activity or levels of astrocyte glutamate transporters (EAAT1 and EAAT2). This is an important point as the weight of evidence suggests that dysfunction and/or downregulated expression of EAAT2, which is responsible for approximately 90% of glutamate reuptake by humans astrocytes, is a major cause, if not the major cause, of glutamate-mediated NMDA receptor excitotoxicity, which appears to be causatively implicated in the pathogenesis and pathophysiology of all neurodegenerative and neuroprogressive disorders [232,233].

The results of human and animal studies point to a major cause of such downregulated expression or dysfunction of these receptors seen in all these illnesses as being the upregulated canonical NF-κB signaling and elevated levels of PICs such as tumor necrosis factor alpha (TNF-α) and interleukin (IL)-1β, ROS and RNS characteristic of the intracellular environment of reactive astrocytes ([234]; reviewed by [233]). TNF-α appears to downregulate the transcription of the *EAAT2* gene [209,235] while IL-1β primarily has a negative effect on the membrane density of the receptor by enhancing its endocytosis and sequestration in the cytoplasm [236,237]. There is also evidence suggesting that the function of EAAT2 (and indeed EAAT1) is compromised in neurodegenerative and neuroprogressive illnesses as a result of S-nitrosylation of crucial thiol residues, which play an indispensable role in their function (reviewed by [238]).

There is robust in vivo evidence that increasing ROS scavenging and GSH production, using *N*-acetylcysteine, can increase EAAT and decrease X_c_^−^ expression in reactive astrocytes [239]. Hence, the consumption of a KD, which also results in increased GSH production in the brain via the upregulation of Nrf2 [43,46] would be expected to have a similar beneficial effect. There are also an increasing number of publications reporting reduced levels of NF-κB and PIC levels in astrocytes and indeed other regions of the brain following ingestion of various manifestations of the KD [1,240] and hence the diet seems to have the capacity to exert a corrective influence on several elements driving the EAAT2 downregulation seen in neurodegenerative and neuroprogressive disorders.

In addition, EAAT transcription is a downstream target of PPAR whose upregulation has a range of neuroprotective effects in neuropathological conditions in vivo [241,242]. This is of particular importance as there are several studies reporting upregulation of PPAR activity following the prolonged ingestion of a KD and this provides another mechanism by which diet-induced ketosis could upregulate the transcription of EAATs in reactive astrocytes [127,131]. In addition, there is an accumulating body of data suggesting that the upregulation of PPAR reduces neuroinflammation, which is of interest because the presence of this phenomenon is a major trigger of increased astrocyte reactivity as discussed above [136]. Furthermore, PPAR activation may rescue the compromised mitochondrial bioenergetics and dynamics seen in diseases such as AD and SZ [137,138].

### Nutritional ketosis, astrogliosis, and NKA function

This is of paramount importance from the perspective of this article. Glutamate and GABA uptake are energy consuming processes as previously discussed [199,243] due in part to the reliance of EAATs on the option function of NKA receptors which coexist in the same molecular complex [244] (reviewed by [243]). This enzyme, as the name suggests, is in turn dependent on adequate supplies of ATP [245] and hence its function is likely to be compromised in an environment of impaired bioenergetics characteristic of astrocytes in their reactive state [55].

Improving the function of NKA is clearly a desirable therapeutic target and in that context, it is important to note that several research teams have reported that protracted, acute, or intermittent ketosis activates or increases the expression NKA pumps in the brain [68,246,247]. It should be stressed that this finding is not only important from the perspective of glutamate homeostasis as the interplay between NKA and EAATs, but also plays an important role in regulating levels of K^+^ ions throughout the CNS [248]. From the perspective of K^+^ homeostasis, however, the weight of evidence suggests NKA is the most important player in this molecular partnership and plays the dominant role in K^+^ uptake into astrocytes, which in turn regulates neural function and excitability [167]. The dependence of K^+^ uptake into astrocytes on NKA activity goes some way to explaining evidence supplied by several authors confirming that the maintenance of K^+^ homeostasis is the most energy intensive role of astrocytes [249] and thus dependent on adequate supplies of ATP [250,251]. The function of NKA and its indispensable role in astrocyte is well documented and hence will not be considered here but any readers interested in a detailed consideration of the biochemistry underpinning its structure and functions are referred to excellent reviews by Roy et al. [245] and Rodrigo et al. [252].

However, from the perspective of this article, it should be emphasized that the activity of NKA is not just dependent on adequate supplies of ATP but is heavily influenced by the redox state of the intracellular and extracellular environment. Unsurprisingly, animal and human studies confirm that oxidative and nitrosative stress inhibits NKA activity in rat hippocampus and prefrontal cortex [253,254] and in patients with SZ and BPD [255,256]. A combination of oxidative stress would also appear to explain the downregulation of NKA activity seen in MDD, AD, and other neurodegenerative diseases (reviewed by [257]). These are important observations as accumulating evidence suggests that downregulation of NKA is partly responsible for the impaired ability of reactive astrocytes to regulate K^+^ homeostasis [211,258].

The causative role played by oxidative and nitrosative stress in NKA downregulation is further emphasized by studies reporting that prolonged use of antioxidant combinations such as vitamin C and E or *N*-acetylcysteine, α-tocopherol, and α-lipoic acid can produce clinically significant increases in NKA activity in the brain and periphery [259,260]. Thus, there is a prospect that the well-documented antioxidant properties of the KD may exert a similar effect assuming a similar effect size and may well underpin the observations reporting a positive effect of the KD on improving NKA function discussed above. However, there may be another mechanism by which nutritional ketosis may improve NKA function, which would appear to be under-discussed.

Briefly, several authors have also reported strong negative correlations between the extent of membrane lipid peroxidation and NKA activity in vivo in illnesses as diverse as cardiovascular disease, SZ, and BPD [255,256,261]. The relationship between increased membrane lipid peroxidation and increasing NKA dysfunction appears to be mediated by decreased membrane fluidity and PUFA content [260,262]. This is of importance as several studies have reported a significant increase in NKA activity following dietary supplementation with PUFAs [260,263–265]. These observations may be explained by reference to data confirming that dietary PUFAs can integrate into lipid membranes in the periphery and the brain combatting the drivers of lipid peroxidation and increasing membrane fluidity via mechanism (reviewed by [266]). In this context, it is noteworthy that the KD can elevate levels of PUFAs in the circulation, cerebrospinal fluid, and brain [267,268] and hence this property may afford yet another route by which diet-induced ketosis might upregulate NKA levels and function. There is also some evidence to suggest that reducing lipid membrane peroxidation and improving membrane stability in astrocytes may help to increase the expression of another receptor, which also plays an indispensable role in K^+^ homeostasis mediated by these glial cells, namely the aforementioned weak inwardly rectifying K_ir_ family containing K_ir_4.1 [269–271]. This is important as the downregulation of this receptor in reactive astrocytes is the other major cause of impaired K^+^ homeostasis in the brain [183,184].

### Nutritional ketosis, astrogliosis, and K_ir_4.1 function

These findings are a reflection of the fact that astrocyte-mediated K^+^ buffering is mainly enabled by the presence of K_ir_ family potassium channels containing K_ir_4.1 and K_ir_4.1/5.1 subunits [272,273]. Much evidence suggests that the K_ir_4.1 is the most important channel in astrocyte-mediated spatial buffering and may be responsible for up to 45% of potassium buffering in the hippocampus [274,275]. Readers interested in regarding the structure and mechanisms underpinning the operation of K_ir_4.1 and other astrocyte K_ir_ family channels are invited to consult the work of Brill et al. [276].

K_ir_4.1 activity has been associated with several other elements involved in astrocyte structure and function such as the regulation of astrocyte cell volume, astrocyte K^+^ conductance, resting membrane potential, and glutamate uptake [277,278]. Importantly, several research teams have reported that inhibition of this channel leads to increased K^+^ concentrations in the extracellular space and impaired glutamate uptake [279,280]. The resultant increase of glutamate in the synaptic cleft resulting from K_ir_4.1 inhibition results in abnormal modulation of synaptic transmission and network level communication [277,281] and is associated with the development and maintenance of neuroinflammation [282,283]. The pathological significance of K_ir_4.1 downregulation in a neuroinflammatory environment is further emphasized by the results of several in vivo studies reporting reduced expression and/or function of this receptor in several neurodegenerative diseases, most notably multiple sclerosis and AD [284–287].

Downregulation of K_ir_4.1 is also seen in patients with MDD [288] and there is some evidence to suggest that reduced K_ir_4.1 expression plays a causative role in the development of SZ and ASD [289].

Given the potential therapeutic importance of improving K_ir_4.1 expression and/or function, it is encouraging to note that there is evidence that some of the elements responsible for the downregulation of astrocytic K_ir_4.1 receptors in an environment of chronic neuroinflammation are very similar if not identical to the drivers of impaired EAAT expression and activity discussed above with increased IL-1 [270,282,290] and glutathionylation [291] playing important inhibitory roles. Hence, the proven capacity of nutritional ketosis to reduce levels of oxidative stress and inflammation in the brain discussed on several occasions above might be expected to improve the expression and function of this receptor assuming that such reductions are of sufficient magnitude needed to produce such an effect. Other factors known to reduce K_ir_4.1 expression in vivo seen in neurodegenerative and neuroprogressive conditions include high levels of extracellular glutamate, which stimulate NMDA receptors located on astrocytes leading to K_ir_4.1 downregulation [292–294].

Hence, improvements in glutamate reuptake by astrocytes coupled with amelioration of glutamate dyshomeostasis, which may stem from nutritional ketosis would be expected to produce concomitant improvements in K_ir_4.1-mediated astrocyte K^+^ buffering. Increased levels of ATP fostered by the bioenergetic and metabolic consequences of induced ketosis may also be of therapeutic benefit as far as improving K^+^ homeostasis mediated by K_ir_4.1 is concerned as the optimum function of this receptor is also dependent on adequate levels of ATP [285,286,295,296]. Consequently, the documented improvements in oxidative stress, neuroinflammation, glutamate homeostasis, and ATP production in the CNS following prolonged ingestion of various KDs would be expected to result in beneficial effects on K_ir_4.1 levels and function.

### Nutritional ketosis, astrogliosis, AQP4, and gap junction function

There are replicated in vivo data confirming that nutritional ketosis and/or BHB administration may upregulate AQP4 activity [70,297] and normalize Cx43 gap junction function [298–300]. This is also important from the perspective of K^+^ homeostasis as the activity of K_ir_4.1 channels in astrocyte-mediated K^+^ buffering is aided by the activity of AQP4 (reviewed by [301]) and astrocyte gap junctions [275].

The expression, structure, and activity of AQP4 is significantly compromised in the environment of chronic neuroinflammation seen in neurodegenerative and neuroprogressive disorders [302,303]. These ROS- and NO-mediated alterations lead to compromised performance of the receptor in regulating K^+^ homeostasis and several other dimensions of CNS homeostasis such as water balance, glutamate uptake, adult neurogenesis, and astrocyte migration (reviewed by [304]). Impaired expression of AQP4 perpetuates and exacerbates neuroinflammation and astrogliosis and this phenomenon also underpins the detrimental role played by this receptor in the pathophysiology of AD, PD, MDD, and ASD [305] (reviewed by [303]).

Astrocytic Cx43 gap junctions and pannexin hemichannels are held in an open configuration in a state of chronic neuroinflammation and oxidative stress as a result of the S-nitrosylation and oxidative modification of regulatory cysteine thiol motifs leading to conformational changes and loss of functional plasticity (reviewed by [306,307]). This compromised function not only impairs their role in K^+^ homeostasis but also negatively impairs astrocyte-mediated glutamate uptake and dispersal [308,309]. There is also evidence that a state of chronically open gap junctions induces and exacerbates neuroinflammation [308,309] (reviewed by [310]).

These consequences would appear to underpin at least in part evidence implicating gap junction and pannexin hemichannel dysfunction as another causative factor in the development of neurodegenerative and neuroprogressive disorders [309,311] (reviewed by [312]).

### Nutritional ketogenesis, astrogliosis, and K_ATP_ channel function

Several animal studies have reported that prolonged ingestion of the KD or administration of the KD results in the opening of K_ir_6.1 family K_ATP_ channels located in cell plasma membranes (sK_ATP_) and in the outer membranes of mitochondria (mtK_ATP_) [44, 313–315]. It could be argued that this effect results from increasing ATP levels, which are increased following ingestion of various KDs as discussed above. However, given the fact that these channels are opened in an environment of low ATP and high ATP [316,317] and the significant increase in ATP production in the brain induced by the KD, it is possible that K_ATP_ opening is mediated by decreasing oxidative stress and neuroinflammation. This argument is strengthened by evidence demonstrating inhibition of K_ATP_ channels by glutathionylation in an environment of increased oxidative stress [309–311, 318].

Irrespective of the mechanisms underpinning such upregulation, however, there is evidence to suggest that increased activity of these channels also has positive consequences for astrocyte function. For example, one such consequence is improved sequestration of K^+^ into mitochondria via a mechanism, which is similar in many respects to the mechanism enabling the sequestration of iron [312,313]. There is also evidence, albeit in vitro, of a positive association between the activation of astrocytic mtK_ATP_ channels and upregulation of electrical coupling between astrocytes in the hippocampus which is an effect mediated via increased efficiency of Cx43 gap junction function secondary to upregulated ERK signaling in astrocytic mitochondria (reviewed by [299]). It is also noteworthy that opening of astrocyte mtK_ATP_ channels may also be important from the perspective of CNS homeostasis as its upregulation appears to be an important element in maintaining the stability of the wider astrocyte neurovascular unit [314].

There are also replicated data suggesting that opening mtK_ATP_ channels may make a significant contribution to astrocyte survival in an environment of chronic inflammation and oxidative stress by inhibiting the translocation of Bax and the release of cytochrome *c* oxidase from mitochondria into the cytosol thereby inhibiting ROS- or TNF-mediated apoptosis [315–317]. Finally, one team of researchers has produced tantalizing evidence of an association between the opening of K_ATP_ channels and the inhibition of astrocyte activation and the prevention of astrogliosis [318].

## Caveats and Uncertainty

Although the data reviewed above suggest that BHB entry into the brain is a major driver of the therapeutic benefits of the KD or of KB supplements, it should be emphasized that other factors may be involved and the mechanisms underpinning such therapeutic benefits are not completely understood either in the case of epilepsy or otherwise. For example, several authors have reported profound changes in the composition of the microbiota following the administration of a KD in children with intractable epilepsy, which appear to be important if not essential for seizure control [231,319,320]. In general, increases in Firmicutes and Actinobacteria are seen in KD-responding children. Although, increases in *Alistipes* and Ruminococcaceae are apparent in nonresponders [319]. Similar patterns have been reported by authors investigating KD effects in animal models of epilepsy, with a positive effect being associated with changes in the composition of the microbiota associated with relative increases in levels of *Akkermansia* and *Parabacteroides* [231]. Data suggesting that fecal transplants based on *Akkermansia* and *Parabacteroides* also exert an antiseizure effect are also of interest [231]. The association between changes in the microbiota and improved seizure control can be understood in the context of accumulating evidence demonstrating that the composition of the microbiota exerts profound effects on metabolism and inflammatory status via numerous mechanisms such as influencing levels of short-chain FAs and intestinal barrier integrity [321,322]. For example, increased levels of *Akkermansia* and *Parabacteroides* increase intestinal barrier integrity via a positive effect on epithelial tight junction proteins and hence reduce the translocation of commensal antigens into the blood, the latter being a powerful driver of peripheral inflammation [323–325]. Elevated levels of BHB also result in the suppression of peripheral inflammation via the inhibition of NF-κB and the NLRP3 inflammasome (reviewed by [1]). This latter point is important because peripheral inflammation is also a driver of pro-inflammatory dysbiosis via mechanisms explained in [326] and [321]. Hence, the reduction in peripheral inflammation seen in individuals following protracted consumption of a KD could potentially explain the positive effects on the composition of the gut population seen above, which in turn could make an independent contribution to the reduction of peripheral inflammation. This is an important point because peripheral inflammation in the guise of elevated PICs is a major driver of microglial and astrocytic activation, proliferation and/or dysfunction [327,328], which are all involved in the development of severe intractable epilepsy [329,330]. Hence, it is tempting to conclude that the reduction in peripheral inflammation explains the positive effects of a KD on seizure control and on the microbiome. However, rodents consuming a KD have an increased GABA/glutamate ratio in their brains, which appears to stem from positive changes to the composition of the microbiota [278]. This observation is supported by other lines of evidence suggesting that manipulation of the gut commensal population can exert positive effects on glutamatergic neurotransmission, which is compromised in patients with intractable epilepsy and neuroprogressive disorders [331–333]. In addition, the reduction of dysbiosis or positive changes to the composition of the microbiota can exert a number of additional neuroprotective effects mediated via the enteric nervous system and the vagus nerve (reviewed by [326]). Thus, the positive effects on seizure control effected by the KD associated with changes in the microbiota are likely underpinned by multifactorial mechanisms.

There is also evidence to suggest that a KD may exert antiseizure and neuroprotective effects by inducing a unique metabolic state of increased serum leptin in combination with reduced serum insulin [334–337]. This state is associated with modification of the PI3k/Akt/mTOR signaling axis and AMPK levels and therefore may be responsible for the reduced levels of mTOR and Akt seen in the hypothalamus following prolonged intake of a KD [338,339]. Increased leptin in the brain may result in improved function of K_ATP_ channels, inhibition of AMPA receptors, and improved function of NMDA receptors via a mechanism dependent on increased PI3K signaling [340,341]. Reduced levels of insulin in the periphery in patients with metabolic syndrome or type 2 diabetes mellitus, frequently seen in patients with neuroprogressive disorders [342], can also exert neuroprotective effects by reducing the translocation of ceramide into the CNS which is often described as the liver–brain axis of neurodegeneration (reviewed by [343]). The range of neuroprotective effects potentially resulting from a metabolic state of increased leptin and reduced insulin in the periphery and the mechanisms involved are numerous and readers interested in pursuing this area are invited to consult an excellent review of the subject by [339].

An attempt at explaining the neuroprotective effects of induced ketosis or ketonemia is further complicated by evidence suggesting that a low glycemic index diet may also exert antiseizure activity [344]. This neuroprotective effect could potentially be explained by reduced insulin and triglyceride levels coupled with improved insulin resistance, which both induce anti-inflammatory effects and hence would be expected to reduce levels of glial cell pathology via mechanisms discussed above [345,346]. However, a perusal of the literature suggests that low glycemic carbohydrates have largely been used in the context of a KD and hence the effectiveness of this approach is difficult to assess [347]. A study comparing the effects on seizure control of a low glycemic diet, which does not involve the induction of ketosis compared with the use of low glycemic carbohydrates in the context of a KD may well provide clarity in this area.

## Conclusions

This article illustrates how the entry of KBs and FAs into the CNS, as a result of a ketotic state resulting from nutritional ketosis, has effects on the astrocytic glutamate–glutamine cycle, GS activity, and on the function of VGLUTs, EAATs, NKA, Kir4.1, AQP4, Cx34, and K_ATP_, as well as on astrogliosis, which would tend to mitigate the changes seen in a wide range of neurodegenerative and neuroprogressive disorders. These disorders include, but are not limited to, AD, PD, HD, SZ, BPD, MDD, and ASD. It is therefore plausible to hypothesize that nutritional ketosis may have therapeutic applications in the treatment of such disorders. However, it must also be stated that the KD results in a number of effects in the periphery such as changes in the composition of the microbiota, and alterations in levels of leptin and insulin, which combined with a reduction of inflammation, may also contribute to the antiseizure and neuroprotective effects of induced ketosis or ketonemia.

## Glossary

ACA: acetoacetate
AD: Alzheimer’s disease
ALS: amyloid lateral sclerosis
AQP4: aquaporin-4
ASD: autistic spectrum disorder
BBB: blood–brain barrier
BDNF: brain-derived neurotrophic factor
BHB: β-hydroxybutyrate
BPD: bipolar disorder
CNS: central nervous system
Cx43: connexin 43
ETC: electron transfer chain
FA: fatty acid
FFA: free fatty acid
GS: glutamate synthetase
GSH: reduced glutathione
HD: Huntington’s disease
KB: ketone body
KD: ketogenic diet
LPS: lipopolysaccharide
MCFA: medium-chain fatty acid
MCT: medium-chain triglyceride
MDD: major depressive disorder
NEFA: nonesterified fatty acid
NKA: Na^+^, K ^+^ -ATPase
PC: pyruvate carboxylase
PD: Parkinson’s disease
PIC: pro-inflammatory cytokine
PUFA: polyunsaturated fatty acid
RNS: reactive nitrogen species
ROS: reactive oxygen species
SIRT: sirtuin
SOD: superoxide dismutase
SZ: schizophrenia
TCA: tricarboxylic acid
VGLUT: vesicular glutamate transporter
X_c_^-^: cysteine/glutamate antiporter channel
